# Supplementary material for: Prevalence of complementary and alternative medicine despite limited perceived efficacy in patients with rheumatic diseases in Mexico: Cross-sectional study
Source: PLoS One. 2021 Sep 28;16(9):e0257319. doi: 10.1371/journal.pone.0257319 (PMC8478211; doi:10.1371/journal.pone.0257319)
Supplement: S2 File — (DOCX) [file pone.0257319.s002.docx]

**Date (__/__/__) Folio:_____ Hospital:______________________**

Dear patient:

This questionnaire is part of the research protocol on the use of alternative and complementary medicine in patients with rheumatic diseases. We ask for your support by answering a series of questions related to your illness and the use of this type of therapy, if you agree it will take approximately 15 minutes of your time, the information you provide will be completely CONFIDENTIAL and ANONYMOUS. We appreciate your valuable participation.

**GENERAL DATA**

1. **Age: _____ 2. Sex:** a. Male b. Female **3. Residence: _____________________**
2. **Scholarship** a. Elementary b. HIghschool c. College d. Master e. PHD d. None
3. **Religion**: ________________ **6.Ethnicity**::_______________
4. **Marital Status:** a. Single b. Married c. Divorced d. Widow
5. **Job:______________________**

**9. Do you use any orthopedic appliance?** a. Yes b. No **10. Which one? _________________________**

1. **Rheumatic disease**
2. **What is your rheumatological diagnosis? ________________________**
3. **When did your symptoms started? __________**
4. **When where you diagnosed? ___________**
5. **Comorbidities**

_____________________ _______________________ ________________________

_____________________ _______________________ ________________________

1. **What medications do you take?**

_____________________ _______________________ ________________________

_____________________ _______________________ ________________________

| 1. **Morisky Medication Adherence Scale (MMAS-4)** | **Yes** | **No** |
| --- | --- | --- |
| Do you ever forget to take your (name of health condition) medicine? |  |  |
| Do you ever have problems remembering to take your (name of health condition) medication? |  |  |
| When you feel better, do you sometimes stop taking your (name of health condition) medicine? |  |  |
| Sometimes if you feel worse when you take your (name of health condition) medicine, do you stop taking it? |  |  |

1. **COMPLEMENTARY AND ALTERNATIVE MEDICINE USE**

**Complementary and alternative medicine (CAM) is the term for medical products and practices that are not part of standard medical care**

1. **Have you ever used any of these therapies to control your RHEUMATIC DISEASE?**
   1. Yesi b. No

**IF THE ANSWER TO THE PREVIOUS QUESTION WAS NO, PLEASE GO TO QUESTION 31**

1. **The catalog of products and alternative therapies will be shown below, with the data collected, please fill in the following table as requested. After showing the catalog, ask if they have used any other alternative treatment that does not appear in the images and include it in the table (It is important to clarify that it is for the treatment of rheumatic disease or its symptoms, not as food or other unrelated disease to the same).**

| **Name of the CAM** | **Have you used it in the last year?** | | **Please indicate the main reason you used it** | | | | | **How much improvement did you find with this product?** | | | | **How did you find out about this product?** | | | | | |
| --- | --- | --- | --- | --- | --- | --- | --- | --- | --- | --- | --- | --- | --- | --- | --- | --- | --- |
|  | **Yes** | **NO** | **Articular pain** | **improve immune system** | **Desinflamar** | **Cure disease** | **Other** | **None** | **Mild** | **Moderate** | **Very** | **Family** | **Friend** | **Internet** | **Neewspaper** | **Physician** | **Other** |
|  |  |  |  |  |  |  |  |  |  |  |  |  |  |  |  |  |  |
|  |  |  |  |  |  |  |  |  |  |  |  |  |  |  |  |  |  |
|  |  |  |  |  |  |  |  |  |  |  |  |  |  |  |  |  |  |
|  |  |  |  |  |  |  |  |  |  |  |  |  |  |  |  |  |  |
|  |  |  |  |  |  |  |  |  |  |  |  |  |  |  |  |  |  |
|  |  |  |  |  |  |  |  |  |  |  |  |  |  |  |  |  |  |
|  |  |  |  |  |  |  |  |  |  |  |  |  |  |  |  |  |  |
|  |  |  |  |  |  |  |  |  |  |  |  |  |  |  |  |  |  |
|  |  |  |  |  |  |  |  |  |  |  |  |  |  |  |  |  |  |
|  |  |  |  |  |  |  |  |  |  |  |  |  |  |  |  |  |  |
|  |  |  |  |  |  |  |  |  |  |  |  |  |  |  |  |  |  |
|  |  |  |  |  |  |  |  |  |  |  |  |  |  |  |  |  |  |
|  |  |  |  |  |  |  |  |  |  |  |  |  |  |  |  |  |  |
|  |  |  |  |  |  |  |  |  |  |  |  |  |  |  |  |  |  |
|  |  |  |  |  |  |  |  |  |  |  |  |  |  |  |  |  |  |
|  |  |  |  |  |  |  |  |  |  |  |  |  |  |  |  |  |  |
|  |  |  |  |  |  |  |  |  |  |  |  |  |  |  |  |  |  |
|  |  |  |  |  |  |  |  |  |  |  |  |  |  |  |  |  |  |

1. **Approximately how much money do you spend a month on alternative products and therapies?____________**
2. **In the last month, how many days have you used any type of product or alternative therapy? (1-31)____________**
3. **How long ago have you been using it?**
   1. Less than a month b. 1-5 Months c. 6 months- 1 year d. More than a year
4. **After using the alternative treatments and therapies, did you have any negative symptoms or reactions?**
   1. Yes b. No
5. **what was the negative reaction or symptom**______________________________________________________________
6. **When you have used alternative therapies, have you informed your rheumatologist? a. Y**es b. No
7. **What was the reason for informing your doctor?**
   1. The doctor must know everything I am taking
   2. The doctor asked me
   3. Know if it has any interaction with the medicine that the doctor prescribes
   4. To ask how alternative therapy works
   5. Other (specify):
8. **What did your doctor advise you about using alternative therapies?**
   1. Do not use b. Use it c. Indifferent
9. **Have you ever suspended the treatment provided by your rheumatologist due to the use of alternative therapies?**
   1. Yes b. No
10. **Do you consider that your disease is under control with the treatment given by your rheumatologist?**
    1. Yes b. No
11. **Which kind of therapy do you trust more?**
    1. Rheumatologist’s b. CAM c. Both
12. **Has the person who gives you CAM ever advised you to abandon the treatment prescribed by your rheumatologist??**
    1. Yes b. No
13. **FAMILY AND SELF-CARE**
14. **Does anyone else in your immediate family have a RHEUMATIC DISEASE?**
    1. Yes b. No
15. **Do they support you in your care process?**
    1. Yes b. No
16. **At some point in your illness, have you needed a caregiver? a. Yes b. No**

**_____________________________________________________________________________________**

1. **Routine Assessment of Patient Index Data (RAPID 3). Please check the ONE best answer for your abilities at this time:**

| **OVER THE LAST WEEK, were you able to:** | **without ANY**  **difficulty** | **with SOME**  **difficulty** | **with MUCH**  **difficulty** | **UNABLE**  **to do** |
| --- | --- | --- | --- | --- |
| **a. Dress yourself, including tying shoelaces and**  **doing buttons?** |  |  |  |  |
| 1. **Get in and out of bed?** |  |  |  |  |
| 1. **Lift a full cup or glass to your mouth?** |  |  |  |  |
| 1. **Walk outdoors on flat ground?** |  |  |  |  |
| 1. **Wash and dry your entire body?** |  |  |  |  |
| 1. **Bend down to pick up clothing from the floor?** |  |  |  |  |
| 1. **Turn regular faucets on and off?** |  |  |  |  |
| 1. **Get in and out of a car, bus, train, or airplane?** |  |  |  |  |
| 1. **Walk two miles or three kilometers, if you wish?** |  |  |  |  |
| 1. **Participate in recreational activities and sports as you would like, if you wish?** |  |  |  |  |
| 1. **Get a good night’s sleep?** |  |  |  |  |
| 1. **Deal with feelings of anxiety or being nervous?** |  |  |  |  |
| 1. **. Deal with feelings of depression or feeling blue?** |  |  |  |  |

1. **How much pain have you had because of your condition OVER THE PAST WEEK? Please indicate below how severe your pain has been:**

**NO PAIN PAIN AS BAD AS IT COULD BE**

0 1 2 3 4 5 6 7 8 9 10

1. **Considering all the ways in which illness and health conditions may affect you at this time, please indicate below how you are doing:**

**VERY WELL VERY POORLY**

0 1 2 3 4 5 6 7 8 9 10
